# Carnosine Protects Macrophages against the Toxicity of Aβ1-42 Oligomers by Decreasing Oxidative Stress

**DOI:** 10.3390/biomedicines9050477

**Published:** 2021-04-26

**Authors:** Giuseppe Caruso, Cristina Benatti, Nicolò Musso, Claudia G. Fresta, Annamaria Fidilio, Giorgia Spampinato, Nicoletta Brunello, Claudio Bucolo, Filippo Drago, Susan M. Lunte, Blake R. Peterson, Fabio Tascedda, Filippo Caraci

**Affiliations:** 1 Department of Drug and Health Sciences, University of Catania, 95125 Catania, Italy; annafidilio@yahoo.it (A.F.); carafil@hotmail.com (F.C.); 2 Department of Life Sciences, University of Modena and Reggio Emilia, 41125 Modena, Italy; cbenatti@unimore.it (C.B.); nbrunello@unimore.it (N.B.); tascedda@unimore.it (F.T.); 3 Centre of Neuroscience and Neurotechnology, University of Modena and Reggio Emilia, 41125 Modena, Italy; 4 Department of Biomedical and Biotechnological Sciences, University of Catania, 95123 Catania, Italy; nmusso@unict.it (N.M.); forclaudiafresta@gmail.com (C.G.F.); giorgiaspampinato@unict.it (G.S.); claudio.bucolo@unict.it (C.B.); fdrago@unict.it (F.D.); 5 Center for Research in Ocular Pharmacology-CERFO, University of Catania, 95125 Catania, Italy; 6 Ralph N. Adams Institute for Bioanalytical Chemistry, University of Kansas, Lawrence, KS 66047-1620, USA; slunte@ku.edu; 7 Department of Pharmaceutical Chemistry, University of Kansas, Lawrence, KS 66047-1620, USA; 8 Department of Chemistry, University of Kansas, Lawrence, KS 66047-1620, USA; 9 Division of Medicinal Chemistry and Pharmacognosy, College of Pharmacy, The Ohio State University, Columbus, OH 43210, USA; peterson.1119@osu.edu; 10 Department of Laboratories, Oasi Research Institute—IRCCS, 94018 Troina, Italy

**Keywords:** carnosine, macrophages, reactive oxygen species, nitric oxide, peroxynitrite, apoptosis, oxidative stress, phagocytosis, Alzheimer’s disease

## Abstract

Carnosine (β-alanyl-L-histidine) is a naturally occurring endogenous peptide widely distributed in excitable tissues such as the brain. This dipeptide has well-known antioxidant, anti-inflammatory, and anti-aggregation activities, and it may be useful for treatment of neurodegenerative disorders such as Alzheimer’s disease (AD). In this disease, peripheral infiltrating macrophages play a substantial role in the clearance of amyloid beta (Aβ) peptides from the brain. Correspondingly, in patients suffering from AD, defects in the capacity of peripheral macrophages to engulf Aβ have been reported. The effects of carnosine on macrophages and oxidative stress associated with AD are consequently of substantial interest for drug discovery in this field. In the present work, a model of stress induced by Aβ1-42 oligomers was investigated using a combination of methods including trypan blue exclusion, microchip electrophoresis with laser-induced fluorescence, flow cytometry, fluorescence microscopy, and high-throughput quantitative real-time PCR. These assays were used to assess the ability of carnosine to protect macrophage cells, modulate oxidative stress, and profile the expression of genes related to inflammation and pro- and antioxidant systems. We found that pre-treatment of RAW 264.7 macrophages with carnosine counteracted cell death and apoptosis induced by Aβ1-42 oligomers by decreasing oxidative stress as measured by levels of intracellular nitric oxide (NO)/reactive oxygen species (ROS) and production of peroxynitrite. This protective activity of carnosine was not mediated by modulation of the canonical inflammatory pathway but instead can be explained by the well-known antioxidant and free-radical scavenging activities of carnosine, enhanced macrophage phagocytic activity, and the rescue of fractalkine receptor CX3CR1. These new findings obtained with macrophages challenged with Aβ1-42 oligomers, along with the well-known multimodal mechanism of action of carnosine in vitro and in vivo, substantiate the therapeutic potential of this dipeptide in the context of AD pathology.

## 1. Introduction

Amyloid beta (Aβ) is a peptide composed of 42 amino acids, often indicated as Aβ1-42, normally present in both the brain and cerebrospinal fluid of humans [1]. This peptide is implicated in the neuropathological hallmarks of Alzheimer’s disease (AD), which include enhanced oxidative stress [2], pronounced inflammation [3], deposition in the brain of Aβ-insoluble aggregates [4,5], and the formation of neurofibrillary tangles due to the aggregation of hyperphosphorylated tau [6]. It is also well-known that Aβ peptide can undergo aggregation through a cascade process, starting with soluble monomers and going through the formation of soluble oligomer intermediates, high molecular weight protofibrils, and mature and insoluble fibrils [7]. Different factors such as pH, concentration, metal ions, oxidative stress, temperature, and ionic strength can affect the aggregation kinetics [8]. Among all Aβ species, oligomers represent the more toxic species [9]; in fact, the toxic potential of the formed aggregates has been shown to be inversely related to the size of the aggregates [10].

Different specialized cell types are involved in the immune response, with brain-resident (microglia) and macrophages representing those primarily activated [11,12]. Microglia and macrophages can be classified as functionally different populations, with the classically activated (or pro-inflammatory) M1 and alternatively activated (or anti-inflammatory) M2 being the most representative [13]. The classically activated population is characterized by the production of pro-inflammatory cytokines as well as reactive oxygen and nitrogen species (ROS and RNS, respectively), whereas the alternatively activated populations release numerous protective and trophic factors and trigger anti-inflammatory and immunosuppressive responses [14]. In pathological conditions characterized by oxidative stress and inflammation [15,16,17,18], it has been observed that over-activation of microglia and macrophages is linked to the marked production of nitric oxide (NO), superoxide, and peroxynitrite, the latter a very reactive and toxic reaction product of NO and superoxide. With specific regard to AD, in addition to the well-known engulfment of Aβ by microglia [19], peripheral infiltrating macrophages have been shown to play a role in Aβ clearance from the brain via its uptake and subsequent degradation [20,21]. Additionally, a defective capacity of peripheral monocytes/macrophages to engulf Aβ has been reported in AD patients [22].

Carnosine is an endogenous dipeptide discovered in Russia by Gulewitsch and Amiradžibi more than 100 years ago (1900) [23]. The synthesis of this naturally occurring dipeptide requires the activity of carnosine synthase, an enzyme able to combine the two amino acids β-alanine and L-histidine through a reaction requiring a magnesium cation (Mg^2+^) and consumption of adenosine triphosphate (ATP) [24,25]. Although more than 99% of carnosine of the body can be found in cardiac and skeletal muscle [26], reaching millimolar (mM) concentrations (up to 20 mM) [27], levels of this dipeptide are also high in other tissues and organs such as the brain (ranging from 0.7 to 2.0 mM) [28]. Carnosine has also been found in the tissues of some invertebrates [29,30]. A number of previously published studies have shown the therapeutic potential of carnosine in pathologies characterized by abnormal protein aggregation, oxidative stress, and inflammation, such as diabetes [31] and AD [32]. Consequently, the well-known antioxidant, anti-inflammatory, and anti-aggregation activities of carnosine have been recently considered for drug discovery processes in neurodegenerative disorders [33]. Additionally, carnosine interacts with specific receptors localized on the cell membrane and modulates macrophage function [34], acting as a stimulator of the cytotoxic and phagocytic activities of these cells [35]. Furthermore, its ability to decrease oxidative stress and inflammation in an in vitro model of amyloid-induced inflammation [36], as well as to modulate NO production and macrophage polarization [37,38], make this dipeptide an attractive pharmacological tool in the context of AD.

In vivo, the administration of carnosine at the dose of 5 mg/day for a total of 6 weeks has been reported to revert oxidative stress and microglial activation in the hippocampus caused by a high-fat diet in a transgenic mouse model of AD [32]. When quantified in the plasma of probable AD subjects [39], the concentration of this dipeptide has been shown to be significantly decreased (by less than half) compared to healthy subjects. More recently the therapeutic potential of carnosine supplementation in combination with its methylated analogue anserine (β-alanyl-1-*n*-methyl-L-histidine) was shown to counteract cognitive decline in AD subjects [40].

In the present study, we first investigated the toxic potential and the pro-apoptotic activity of Aβ1-42 oligomers in the absence or presence of carnosine. We conducted these studies in macrophages (RAW 264.7 cells) as a well-known and established in vitro model of production of ROS and RNS and related oxidative stress [41,42,43,44,45]. Additionally, to elucidate molecular mechanisms underlying the protective effect of carnosine, we studied the variation of the production of key elements related to oxidative/nitrosative stress, namely NO, total ROS, and peroxynitrite in RAW 264.7 macrophages challenged with Aβ1-42 oligomers in the absence or presence of carnosine. We also investigated the modulation of the expression of selected targets belonging to the canonical inflammatory and oxidative pathways as well as the expression of the genes encoding antioxidant enzymes under the same experimental conditions. In the present manuscript, we show that carnosine counteracts death of macrophages and apoptosis induced by Aβ1-42 oligomers by reducing oxidative stress and negatively modulating the levels of reactive species. This protective activity was also sustained by an enhanced phagocytic activity and the rescue of fractalkine receptor CX3CR1.

## 2. Materials and Methods

### 2.1. Materials and Reagents

RAW 264.7 cells (ATCC^®^ TIB-71™), fetal bovine serum (FBS), Dulbecco’s modified Eagle’s medium (DMEM), trypsin-EDTA solution (0.25% trypsin/0.53 mM EDTA in Hanks’s balanced salt solution (HBSS) without calcium or magnesium), and penicillin/streptomycin antibiotic solution were supplied by American Type Culture Collection (ATCC, Manassas, VA, USA). Centrifuge tubes equipped with 3 kDa molecular weight cut-off filters and water, methanol, far-UV acetonitrile, and chloroform were purchased from VWR International (West Chester, PA, USA). A C-Chip disposable hemocytometer was obtained from Bulldog Bio, Inc. (Portsmouth, NH, USA). HFIP-treated amyloid-peptide (1-42) was obtained from Bachem Distribution Services GmbH (Weil am Rhein, Germany). Sylgard 184 polydimethylsiloxane (PDMS) prepolymer and curing agent, used for the preparation of the hybrid microfluidic devices, were purchased from Ellsworth Adhesives (Germantown, WI, USA). Tentagel M NH_2_ microspheres were obtained from Rapp Polymere GmbH (Tübingen, Germany). Rabbit IgG anti- 2,4-dinitrophenol (DNP) antibody (SP-0603-1) was purchased from Vector Laboratories Inc. (Burlingame, CA, USA). All the remaining materials, unless specified otherwise, were supplied by Sigma-Aldrich Corporate (St. Louis, MO, USA) or Thermo Fisher Scientific Inc. (Pittsburgh, PA, USA).

### 2.2. Preparation of Ab1-42 Oligomers

Synthetic human Aβ1-42 oligomers were prepared according to a validated protocol previously described in detail [36,46]. Briefly, the lyophilized HFIP-treated Aβ1-42 (monomeric form) was first suspended in dimethyl sulfoxide (DMSO) (final concentration of 5 mM) and then diluted by using ice-cold cell culture medium DMEM/F12 (1:1) (final concentration of 100 μM). During the following step, the Aβ1-42 samples were incubated for 72 h at a constant temperature of 4 °C. At the end of this incubation step, Aβ1-42 samples (oligomeric form) were immediately used for the cell treatment or aliquoted and stored at −20 °C until their use.

### 2.3. Cell Maintenance, Propagation, and Treatment Protocol

RAW 264.7 cells were cultured in DMEM enriched with supplements and maintained in 25 or 75 cm^2^ polystyrene cell culture flasks with a vent cap as previously described [42]. In order to avoid overgrowth, we passaged RAW 264.7 cells every 2 to 3 days depending on their confluence. On the day of the experiment, cells were harvested by using a cell scraper, 10 µL of the cell suspension was loaded on a C-Chip for cell counting, and an appropriate number of cells was plated in polystyrene culture flasks in 6- or 96-well plates.

For all the experiments, RAW 264.7 cells were treated for 24 h with Aβ1-42 oligomers (2 µM) (or antibody-bound tentagel beads to measure the phagocytic activity), in the absence or presence of carnosine (20 mM; 1 h pre-treatment). The selection of this Aβ concentration was based on several factors: (i) the goal of achieving significant cell activation and response according to previous validation in a recent work performed in the same cell type [47]; (ii) the ability of this concentration (or even a lower one) to give the same extent of toxicity in highly vulnerable primary neuronal cultures, e.g., mixed [36] or pure [48] neuronal cultures; (iii) the use of Aβ oligomers in an order of magnitude mimicking the transition from physiological (pM to nM levels) to pathological (low µM) conditions. Carnosine pre-treatment at the concentration of 20 mM for 1 h in RAW 264.7 macrophages represents a well-established protocol [37,45,49,50]. As previously reported, carnosine pre-treatment ensures a substantial uptake of carnosine before the stimulation with stressing agents; in fact, carnosine is readily taken up by RAW 264.7 macrophages in cell culture [51].

### 2.4. Analysis of Cell Death

The number of dead cells under the experimental conditions was determined by using a trypan blue exclusion assay as previously described [38]. Each cell suspension was diluted 1:1 to 1:3 (depending on cell density) with 0.4% trypan blue solution and loaded on a C-Chip for the analysis. Live cells, possessing intact cell membranes, excluded the trypan blue dye, whereas dead cells did not. Viable cells were characterized by a clear cytoplasm and dead cells by a blue cytoplasm.

### 2.5. Assessment of Apoptosis and Necrosis

The discrimination between live, necrotic, and apoptotic cells was performed with a FlowSight^®^ (Amnis^®^ FlowSight^®^ Millipore, Merck KGaA, Darmstadt, Germany) imaging flow cytometer. The Violet Chromatin Condensation/Dead Cell Apoptosis Kit was used as a fluorescence assay to reveal the compacted state of chromatin in apoptotic cells according to the manufacturer’s recommendations. The kit contains the cell-permeable Vybrant^®^ DyeCycle™ Violet dye that stains condensed chromatin of apoptotic cells more brightly than chromatin of normal cells and the impermeable red-fluorescent SYTOX^®^ ADvanced™ stain that labels only necrotic cells, based on membrane integrity.

The day prior to treatment, cells were seeded in 6-well plates at the density of 1.5 × 10^6^ cells per well and incubated in a humidified environment (5% CO_2_, 37 °C) to allow complete cell attachment. Cells were left untreated (control) or treated with Aβ1-42 oligomers (2 µM) in the absence or in the presence of carnosine (20 mM; 1 h pre-treatment) for 24 h. At the end of the treatment, the adherent cells were washed in HBSS, trypsinized (trypsin-EDTA solution), and centrifuged at 300× *g*. The cell density was adjusted to ≈1 × 10^6^ cells/mL in HBSS. Each cell suspension (1 mL) was added of 1 μL of 1 μM Vybrant^®^ DyeCycle™ Violet stain and 1 μL of the 500 μM SYTOX^®^ ADvanced™ DMSO solution. Cell suspensions were mixed well and incubated on ice, protected from light, for 30 min. Immediately after the incubation period, the stained cells were analyzed by using 405/488 nm dual excitation. A linearly polarized 785 nm laser was used to measure side scatter. During the acquisition of samples, we were able to remove debris and doublets as well as to select only single cells, creating a scatter plot of area vs. aspect ratio. At least 5–10 × 10^4^ images were collected and about 10,000 events of single cells per sample were acquired.

According to this analysis, we chose 2 revelation channels to identify the different populations on the basis of fluorescence intensity. Each sample can contain 3 populations: live cells showing a low level of violet fluorescence, apoptotic cells showing a higher level of violet fluorescence, and necrotic cells showing violet and red fluorescence. For apoptotic analysis, a scatter plot of the intensity of Vybrant^®^ DyeCycle fluorescence vs. intensity of SYTOX^®^ ADvanced™ fluorescence was added to the analysis area to distinguish the different populations.

### 2.6. Intracellullar NO and ROS Levels Determination

On the day of the experiment RAW 264.7 cells were harvested, counted, plated at a density of 3 × 10^6^ cells/flask, and incubated in a humidified environment (5% CO_2_, 37 °C) to allow for the complete cell attachment. Cells were left untreated (control) or treated with Aβ1-42 oligomers (2 µM) in the absence or in the presence of carnosine (20 mM; 1 h pre-treatment) for 24 h. After 24 h incubation with the treatments, the intracellullar NO and ROS levels in RAW 264.7 cells were determined by using microchip electrophoresis with laser-induced fluorescence (ME-LIF) and 4-amino-5-methylamino-2′,7′-difluorofluorescein diacetate (DAF-FM DA) (in the case of intracellular NO measurement) [37] or 2′,7′-dichlorodihydrofluorescein diacetate (H_2_DCFDA) (in the case of intracellular ROS measurement) [38] probes, as previously described, with slight modifications. Briefly, in each flask, the medium was removed, and cells were washed with phosphate-buffered saline (PBS) and incubated with phenol red-free RPMI-1640 containing DAF-FM DA or H_2_DCFDA (both probes at the final concentration of 10 μM) for 1 h (37 °C, 5% CO_2_). In order to minimize the photobleaching of the probes, during this incubation step, we always covered the flasks with aluminum foil. Next, the medium was removed, and cells were washed with PBS and harvested using a cell scraper. Cells were then counted and centrifuged, and the obtained cell pellet was prepared and analyzed by ME-LIF as recently described [36]. An aliquot of each cell suspension was used for cell counting.

The procedure followed for the fabrication of the disposable hybrid PDMS–glass microchips needed to carry out the ME-LIF experiments has been described previously in detail [42,51,52].

### 2.7. Measurement of Peroxynitrite Generation

The day prior to treatment, cells were seeded in 96-well plates at a density of 2 × 10^4^ cells per well and incubated in a humidified environment (5% CO_2_, 37 °C) to allow for complete cell attachment. Cells were left untreated (control) or treated with Aβ1-42 oligomers (2 µM) in the absence or in the presence of carnosine (20 mM; 1 h pre-treatment) for 24 h. The production of peroxynitrite under all our experimental conditions was carried out by using a recently designed and optimized probe for peroxynitrite detection, Peroxynitrite Sensor 3 (PS3) [52,53]. During the 24 h treatment, PS3 was added to the medium at the final concentration of 10 µM [52]. Wells containing PS3 in absence of cells were used as a negative control. At the end of the treatment, the fluorescence of the plate was analyzed using a Packard Fusion Universal Microplate Analyzer (excitation filter: 485 nm; emission filter: 530 nm) (Packard BioScience Company, Meriden, CT, USA).

Representative images showing the intracellular levels of peroxynitrite in live cells left untreated (control) or treated with Aβ1-42 oligomers (2 µM) in the absence or in the presence of carnosine (20 mM; 1 h pre-treatment) for 24 h were obtained by using a Leica DMI4000 B fluorescence microscope (Leica Microsystems, Wetzlar, Germany) and the Leica LAS AF Lite software (Leica Microsystems).

### 2.8. Total RNA Extraction, Reverse Transcription, and Quantitative Real-Time PCR (qRT-PCR)

The day prior to treatment, cells were seeded in 6-well plates at the density of 1.3 × 10^6^ cells per well and incubated in a humidified environment (5% CO_2_, 37 °C) to allow for complete cell attachment. Cells were left untreated (control) or treated with Aβ1-42 oligomers (2 µM) in the absence or in the presence of carnosine (20 mM; 1 h pre-treatment) for 6 or 24 h. RNA extraction and DNAse treatment were performed as previously described using GenElute™ Mammalian Total RNA Miniprep Kit and DNASE70-On-Column DNase I Digestion Set (Merck KGaA, Darmstadt, Germany) [54,55]. Two micrograms of total RNA were reverse-transcribed with a High-Capacity cDNA Reverse Transcription Kit. Real-time PCR was performed in Bio-Rad CFX Connect thermocycler (Bio-Rad Laboratories, Hercules, CA, USA) using Bio-Rad SsoAdvanced Universal SyBR Mix (Bio-Rad Laboratories) and specific forward and reverse primers at a final concentration of 300 nM (see Table S1 for primer sequences).

The program consisted of 95 °C for 30 s, 40 cycles of 95 °C for 15 s and 60 °C for 30 s. Cycle threshold (Cq) value was determined by the CFX Maestro software (Bio-Rad Laboratories). Glyceraldehyde-3-phosphate dehydrogenase (GAPDH) and cyclophilin A (CypA) were selected as the most stable combination of reference genes using NormFinder [56]. As calibrator, the geometric mean of their Cqs was used [57]. For an appropriate application of the comparative ΔΔCt method, we demonstrated that amplification efficiency of the target and reference genes were approximately equal. For quantitative evaluation of changes, the comparative ΔΔCt method was performed, using as calibrator the average levels of expression of resting cells at 6 and 24 h.

### 2.9. Measurement of Macrophage Phagocytic Activity

Measurement of phagocytic activity in macrophages was carried out through applying a recently developed protocol that allows to measure phagocytosis in RAW 264.7 cells by using antibody-bound tentagel beads and PS3 [52,53].

The antibody-bound tentagel beads were prepared as previously described [52], with only slight modifications. Briefly, tentagel beads were weighed (20 mg), added to 1 mL of PBS, and shacked for 1 h under agitation. At the end of this step, beads were washed twice by using PBS, and then 0.5 mL of PBS and a solution of *N*-succinimidyl *N*-(2,4-dinitrophenyl)-6-aminocaproate (DNP-X-NHS, 5 μL, 50 mM in DMSO) were added. The resulting solution was well mixed with a pipette and shacked for 1 h. After 2 washing steps with ethanol, 300 μL of PBS was added, and 50 μL of this solution was transferred to a new microcentrifuge tube, added of 50 μL of rabbit IgG anti-DNP antibody, and shacked for 1 h. The rest of the beads were stored at 4 °C for later use (used within 2 days to maximize activity). Beads were counted by using a 96-well plate and a CytoFLEX S Flow Cytometer (Beckman Coulter, Brea, CA, USA).

The day prior to treatment, cells were seeded in 96-well plates at the density of 2 × 10^4^ cells per well and incubated in a humidified environment (5% CO_2_, 37 °C). Cells were left untreated (control) or treated with antibody-bound tentagel beads (40000/well) in the absence or in the presence of carnosine (20 mM; 1 h pre-treatment) for 24 h. During the 24 h treatment, PS3 was added to the medium at the final concentration of 10 µM [52]. Wells containing PS3 in the absence of cells were used as a negative control. At the end of the treatment, the cellular fluorescence was analyzed by using a Packard Fusion Universal Microplate Analyzer (Excitation Filter: 485 nm; Emission filter: 530 nm) (Packard BioScience Company).

### 2.10. Statistical Analysis

Graphpad Prism (Ver. 8, San Diego, CA, USA) or SPSS for Windows^®^ v.26 (SPSS Inc., Chicago, IL, USA) software was used for statistical analysis. For multiple comparisons, one-way ANOVA followed by Tukey’s post hoc test was employed. For all the experiments, the statistical significance was set up at *p*-values lower than 0.05.

With regard to relative gene expression, data were analyzed separately for each exposure time (6 and 24 h) with one-way ANOVA followed by Tukey’s post hoc test. Extreme outliers were excluded prior to statistical analysis using the boxplot tool in SPSS (more than 3× the interquartile range outside of the end of the interquartile box).

## 3. Results

### 3.1. Carnosine Protected Macrophages against the Toxicity Induced by Aβ1-42 Oligomers

**Figure 1:**
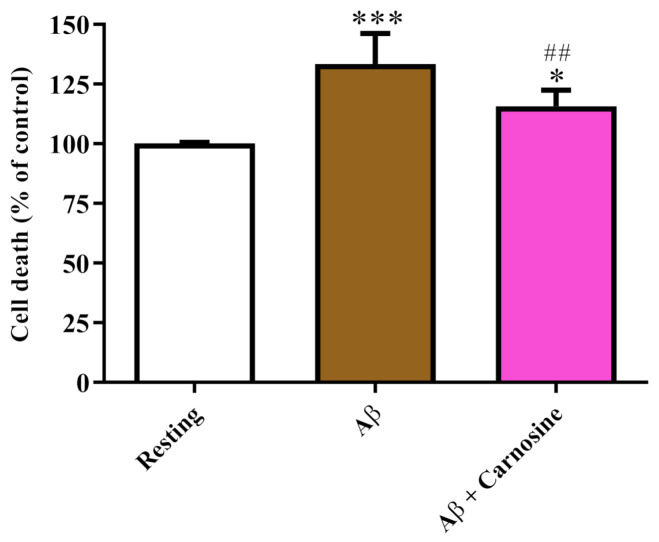
Change in the cell death caused by challenging RAW 264.7 cells with Aβ1-42 oligomers and protective effects of carnosine. RAW 264.7 cells were treated for 24 h with Aβ1-42 oligomers (2 µM) in the absence or presence of carnosine (20 mM; 1 h pre-treatment). Data are the mean of 6 independent experiments and are expressed as the percent variation with respect to the cell death recorded in resting (control) cells. Standard deviations are represented by vertical bars. * significantly different from resting cells, *p* < 0.05; *** significantly different from resting cells, *p* < 0.001; ^##^ significantly different from Aβ1-42 oligomer-treated cells, *p* < 0.01.

Before monitoring the potential protective effects of carnosine, we first investigated the effects of Aβ1-42 oligomers on macrophage cell death. The data depicted in Figure 1 show that the treatment of RAW 264.7 cells with Aβ1-42 oligomers for 24 h significantly increased the number of dead cells (+33%) compared to resting cells (*p* < 0.001).

The cell death induced in RAW 264.7 cells by Aβ1-42 oligomers was significantly counteracted by the presence of carnosine (*p* < 0.01 compared to Aβ1-42 oligomers). Despite the significant protection exerted by carnosine, its presence did not completely abolish the ability of Aβ1-42 oligomers to kill macrophage cells, showing cell death percentage values higher than those observed in resting cells (*p* < 0.05).

### 3.2. Carnosine Inhibited the Pro-Apoptotic Effects Induced by Aβ1-42 Oligomers in Macrophages

After demonstrating the ability of carnosine to counteract the death of macrophages induced by Aβ1-42 oligomers, we evaluated whether the protective effect of this dipeptide was related, at least in part, to its ability to modulate the percentage of cell population undergoing apoptosis.

**Figure 2:**
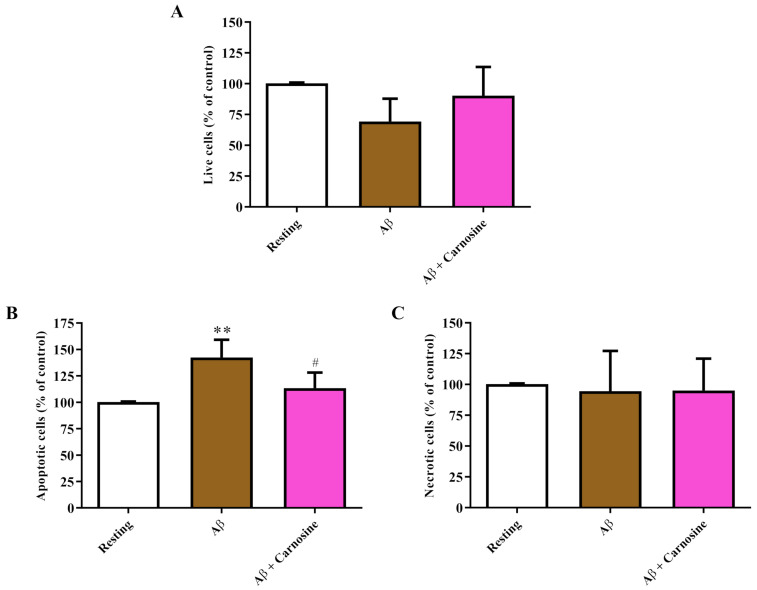
Change in the percentage (%) of (**A**) live cells, (**B**) apoptotic cells, and (**C**) necrotic cells caused by challenging RAW 264.7 cells with Aβ1-42 oligomers and protective effects of carnosine. RAW 264.7 cells were treated for 24 h with Aβ1-42 oligomers (2 µM) in the absence or presence of carnosine (20 mM; 1 h pre-treatment). Data are the mean of 3 independent experiments and are expressed as the percent variation with respect to the number of apoptotic or necrotic cells recorded in resting (control) conditions. Standard deviations are represented by vertical bars. ** significantly different from resting cells, *p* < 0.01; ^#^ significantly different from Aβ1-42 oligomer-treated cells, *p* < 0.05.

The data illustrated in Figure 2 show that treatment for 24 h with Aβ1-42 oligomers induced pro-apoptotic effects in RAW 264.7 cells (+37%; *p* < 0.01 compared to resting cells) (Figure 2B).

The percentage of apoptotic RAW 264.7 cells was significantly decreased in the presence of carnosine (−25%; *p* < 0.05 compared to treatment with Aβ1-42 oligomers) (Figure 2B). An opposite trend, even though not significant, was observed in the case of live cells (Figure 2A), whereas, as expected, no significant changes among all the treatments were found in the case of necrosis (Figure 2C). These results significantly strengthen the protective potential of carnosine shown in Figure 1. In fact, it is worth underlining that the anti-apoptotic effects of carnosine refer to the cells who survived 24 h after the treatment with Aβ1-42 oligomers. This was because the already dead cells, previously identified by trypan blue exclusion test, were not taken into account in this analysis.

### 3.3. Carnosine Decreased Aβ1-42-Induced NO Production in Macrophages

**Figure 3:**
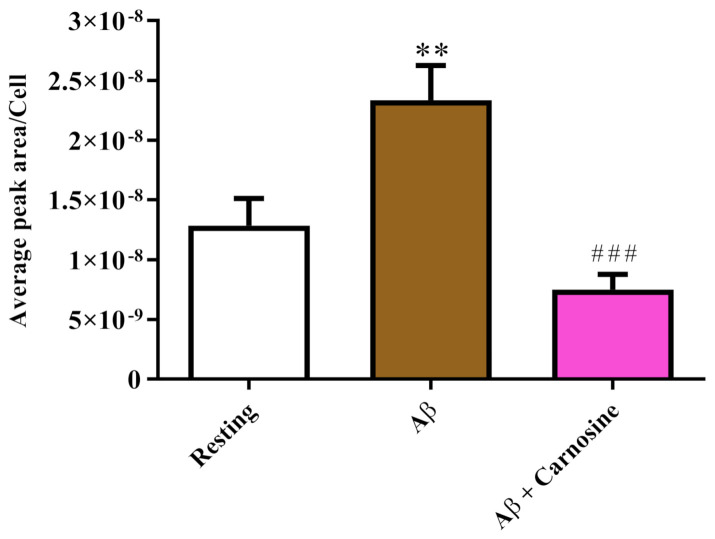
Detection of intracellular concentrations of NO (expressed as average peak area per cell) in resting RAW 264.7 cells and in RAW 264.7 cells stimulated with Aβ1-42 oligomers (2 µM) in the absence or presence of carnosine (20 mM; 1 h pre-treatment). Values are means ± SD of 3 independent experiments. Standard deviations are represented by vertical bars. ** significantly different from resting cells, *p* < 0.01; ^###^ significantly different from Aβ1-42 oligomer-treated cells, *p* < 0.001.

Figure 3 shows the effect of Aβ1-42 oligomers on intracellular NO production in RAW 264.7 cells.

The increase in intracellular NO levels was significant in the case of treatment with Aβ1-42 oligomers (*p* < 0.01 compared to resting cells). To test the effect of carnosine on NO production in stimulated RAW 264.7 cells, we added carnosine 1 h prior to the treatment with Aβ1-42 oligomers. As shown in Figure 3, intracellular NO levels enhanced by treatment with Aβ1-42 oligomers were significantly lowered by the presence of carnosine (*p* < 0.001).

### 3.4. Carnosine Decreased Aβ1-42-Induced Total ROS Production in Macrophages

**Figure 4:**
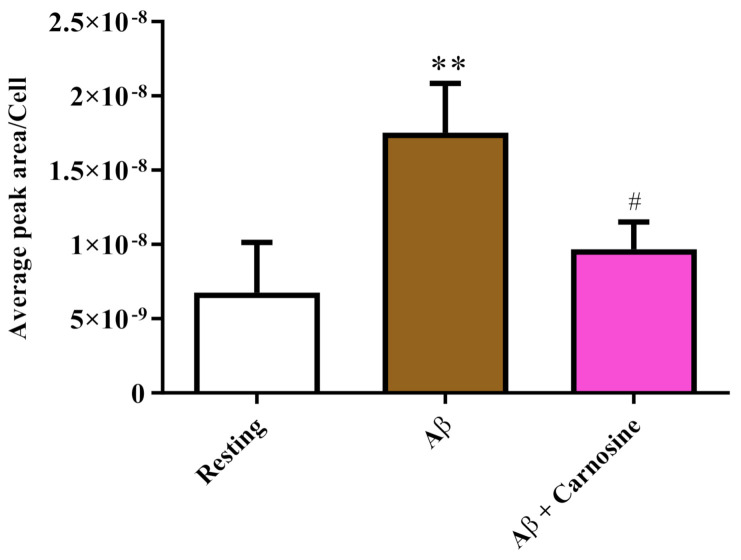
Detection of intracellular concentrations of ROS (expressed as average peak area per cell) in resting RAW 264.7 cells and in RAW 264.7 cells stimulated with Aβ1-42 oligomers (2 µM) in the absence or presence of carnosine (20 mM; 1 h pre-treatment). Values are means ± SD of three independent experiments. Standard deviations are represented by vertical bars. ** significantly different from resting cells, *p* < 0.01; # significantly different from Aβ1-42 oligomer-treated cells, *p* < 0.05.

Figure 4 depicts the effects of treatment with Aβ1-42 oligomers on intracellular ROS production in RAW 264 cells.

A trend quite similar to that seen in the case of intracellular NO was observed during the measurement of total ROS. In fact, as shown in Figure 4, the levels of intracellular ROS were significantly increased by the treatment with the Aβ1-42 oligomers for 24 h (*p* < 0.01 compared to the control). The production of total ROS induced by Aβ1-42 oligomers was significantly decreased by the presence of carnosine (*p* < 0.05 compared to treatment with Aβ1-42 oligomers), giving values comparable to those measured in control cells.

### 3.5. Carnosine Decreased Aβ1-42-Induced Peroxynitrite Production in Macrophages

**Figure 5:**
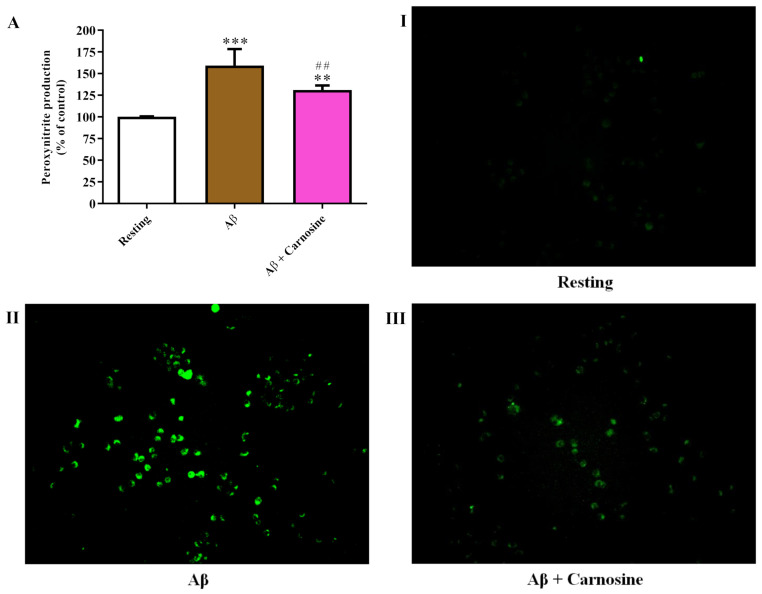
(**A**) Measurement of production of peroxynitrite in resting RAW 264.7 cells and in RAW 264.7 cells stimulated with Aβ1-42 oligomers (2 µM) in the absence or presence of carnosine (20 mM; 1 h pre-treatment). Data are the mean of 4 to 6 independent experiments and are expressed as the percent variation in production of peroxynitrite compared to resting (control) cells. Standard deviations are represented by vertical bars. (**I**–**III**) show representative images of live cells under the indicated treatments obtained by fluorescence microscopy. Images were acquired at 10× magnification. ** significantly different from resting cells, *p* < 0.01; *** significantly different from resting cells, *p* < 0.001; ^##^ significantly different from Aβ1-42 oligomers-treated cells, *p* < 0.01.

An additional set of experiments was carried out to more deeply investigate the pro-oxidant effects of treatment with Aβ1-42 oligomers as well as the ability of carnosine to decrease the levels of reactive species, particularly the formation of the most dangerous of these species, peroxynitrite. Figure 5 shows how treatment with Aβ1-42 oligomers significantly induced the production of peroxynitrite in RAW 264.7 cells compared to resting cells (*p* < 0.001).

As expected, given the results obtained by measuring intracellular NO and total ROS levels, the production of peroxynitrite induced by Aβ1-42 oligomers was significantly increased compared to resting cells (*p* < 0.001). The pre-treatment with carnosine significantly counteracted the ability of Aβ1-42 oligomers to induce peroxynitrite production (*p* < 0.01). Despite the significant antioxidant activity exerted by carnosine, its presence did not completely reduce the levels of peroxynitrite at values comparable to that observe in resting cells (*p* < 0.01).

### 3.6. Carnosine Protective Activity Was Not Mediated through the Canonical Inflammatory Pathway

The murine RAW 264.7 macrophage cell line is often used as a model for inflammatory cells [41,42,43,44,45,58,59]. It appeared that the expression levels of *IL-6* and *IFN-γ* were below detection limit in resting cells, whereas *IL-1β* and more evidently *TNF-α* appeared to be expressed at basal levels (Table S1). Given that stimulation with lipopolysaccharide (LPS) is known to produce a strong inflammatory response in RAW 264.7 cells [60], we first measured the expression levels of immune-related targets (*IL-1β*, *IL-6*, *TNF-α*, *TGF-β1*, *CXCL2*) in cells exposed to LPS (100 ng/mL) and we detected a strong upregulation following a 6 h exposure to LPS that, albeit reduced, was still present after a 24 h exposure to the endotoxin, with the sole exception of *IFN-γ* that remained undetected (Figure S1 and Supplementary Results 1).

**Figure 6:**
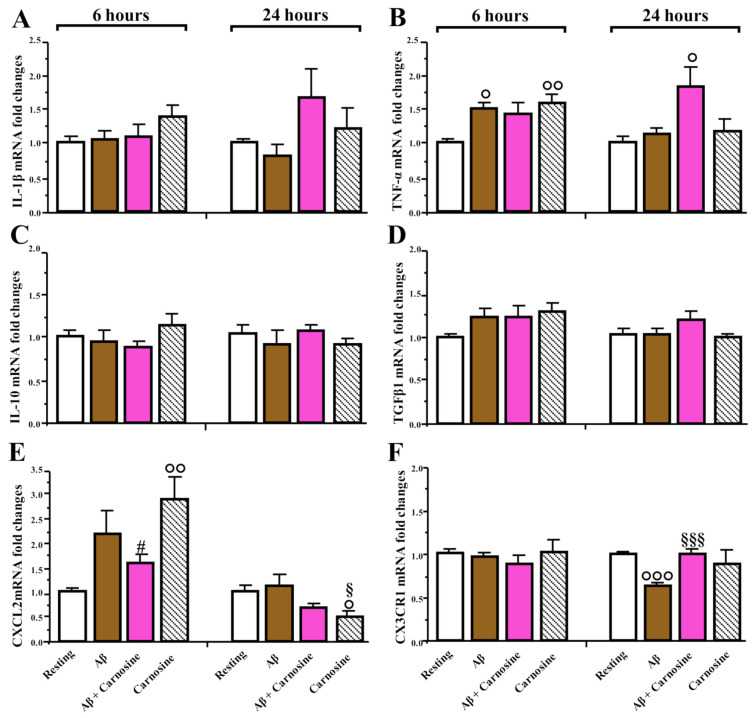
Effects of exposure of RAW 264.7 cells to Aβ1-42 oligomers (2 µM) for 6 and 24 h in the absence or presence of carnosine (20 mM; 1 h pre-treatment) on expression levels of immune-related targets. mRNA expression of (**A**) *interleukin (IL)-1β*, (**B**) *tumor necrosis factor (TNF)-α*, (**C**) *IL-10*, (**D**) *transforming growth factor (TGF)-β1*, (**E**) *macrophage inflammatory protein-2 (CXCL2)*, and (**F**) *CX3CR1*, with *GAPDH*/*CypA* as endogenous controls, was measured by qRT-PCR. Data are represented as means (*n* = 8–12) ± S.E.M. ° significantly different from resting cells, *p* < 0.05; °° significantly different from resting cells, *p* < 0.01; °°° significantly different from resting cells, *p* < 0.001; ^§^ significantly different from Aβ1-42 oligomer-treated cells, *p* < 0.05; ^§§§^ significantly different from Aβ1-42 oligomer-treated cells, *p* < 0.001; ^#^ significantly different from carnosine alone, *p* < 0.05.

In our experimental conditions, exposure to Aβ1-42 oligomers, in the absence or presence of carnosine, or carnosine alone for 6 or 24 h did not affect mRNA levels encoding *IL-1β* (Figure 6A), whereas *IL-6* expression levels remained close to undetectable in all experimental groups (Cq > 35, data not shown).

One-way ANOVA revealed a main effect of treatment for *TNF-α* at both time points. Exposure to Aβ1-42 oligomers or to carnosine alone for 6 h significantly increased mRNA levels coding for *TNF-α* above those of resting cells (of about 50%) (*p* < 0.05 and *p* < 0.01 for Aβ1-42 oligomers and carnosine alone, respectively; Figure 6B). At 24 h instead, a significant increase of about 80% was observed only in cells treated with Aβ1-42 oligomers in the presence of carnosine with respect to resting cells (*p* < 0.05; Figure 6B). Exposure of RAW 264.7 cells to Aβ1-42 oligomers for 6 or 24 h, in the absence or presence of carnosine, or treatment with carnosine alone, did not affect mRNA levels encoding *IL-10* and *TGF-β1* (Figure 6C,D).

The murine functional homologs of human *CXCL8* (IL-8) were differentially expressed in RAW 264.7 cells: *CXCL1* (KC) expression in resting cells remained close to undetectable (Table S1), whereas *CXCL2* (macrophage inflammatory protein-2, MIP-2) mRNA levels were abundantly expressed in untreated cells. The expression levels of *CXCL2* were significantly increased following a 6 h exposure to carnosine alone compared to resting cells (*p* < 0.01) or cells exposed to Aβ1-42 oligomers in the presence of carnosine (*p* < 0.05) (Figure 6E). This effect was reversed after a 24 h incubation: *CXCL2* mRNA levels in cells exposed to carnosine alone were significantly lower (by about 50%) compared to resting cells (*p* < 0.05) or cells exposed to Aβ1-42 oligomers (*p* < 0.05), whereas a similar but not statistically meaningful trend was observed in cells exposed to Aβ1-42 oligomers in the presence of carnosine.

We further measured the expression levels of *CX3CR1*, one of the most highly expressed genes in microglia in mice and humans, implicated in numerous microglial and macrophage functions [61,62]. For this gene, we observed a significant downregulation of expression in RAW 264.7 cells after 6 h exposure to LPS that returned to basal levels after 24 h (Figure S1 and Supplementary Results 1). In our experimental condition, no effect was observed after a 6 h exposure (Figure 6F), whereas exposure to Aβ1-42 oligomers for 24 h caused a significant decrease in *CX3CR1* mRNA levels with respect to resting cells (by about 40%) (*p* < 0.001); this effect was counteracted by pre-treatment with carnosine (*p* < 0.001 compared to Aβ1-42 oligomer-treated cells; Figure 6F).

Activation of RAW 264.7 cells with LPS led to potent upregulation of *inducible nitric oxide synthase (NOS2)* and *prostaglandin-endoperoxide synthase 2 (PTGS2)* after a 6 h exposure (of about 500- and 1500-fold, respectively), which remained at the same order of magnitude after 24 h (Supplementary Results 1).

**Figure 7:**
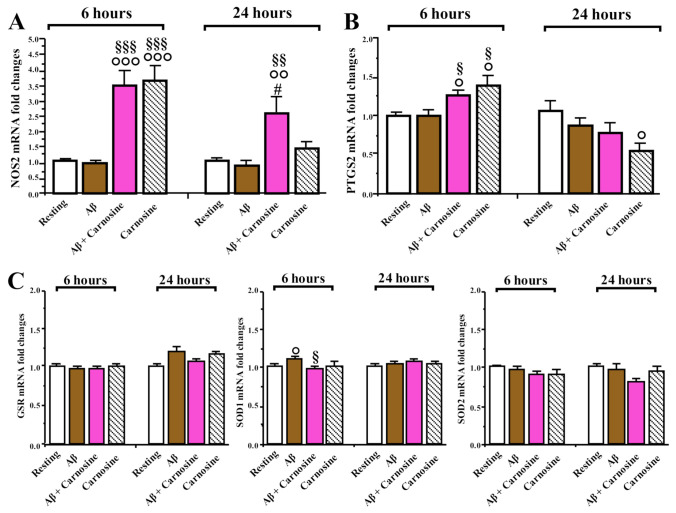
Effects of exposure of RAW 264.7 cells to Aβ1-42 oligomers (2 µM) for 6 and 24 h in the absence or presence of carnosine (20 mM; 1 h pre-treatment) on expression levels of enzymes related to inflammation and oxidative stress. mRNA expression of (**A**) *inducible nitric oxide synthase (NOS2)*, (**B**) *prostaglandin-endoperoxide synthase 2 (PTGS2)*, (**C**) *glutathione reductase (GSR)*, and *superoxide dismutase (SOD) 1 and 2*, with *GAPDH*/*CypA* as endogenous control, was measured by qRT-PCR. Data are represented as means (*n* = 8–12) ± S.E.M. ° significantly different from resting cells, *p* < 0.05; °° significantly different from resting cells, *p* < 0.01; °°° significantly different from resting cells, *p* < 0.001; ^§^ significantly different from Aβ1-42 oligomer-treated cells, *p* < 0.05; ^§§^ significantly different from Aβ1-42 oligomer-treated cells, *p* < 0.01; ^§§§^ significantly different from Aβ1-42 oligomer-treated cells, *p* < 0.001; ^#^ significantly different from carnosine alone, *p* < 0.05.

Incubation for 6 h with carnosine (alone or in combination with Aβ1-42 oligomers) resulted in a significant increase (by about 250%) in the expression of *NOS2* compared to resting cells or cells exposed to Aβ1-42 oligomers (*p* < 0.001; Figure 7A); this effect was reduced, but still present, at 24 h only in the case of cells exposed to Aβ1-42 oligomers in the presence of carnosine (*p* < 0.01 compared to resting cells or Aβ1-42 oligomer-treated cells, *p* < 0.05 compared to carnosine alone). Interestingly, *NOS1* mRNA was undetectable in this cell line (data not shown).

After 6 h of incubation, we observed a significant upregulation of *PTGS2* mRNA levels in cells exposed to carnosine (alone or in combination with Aβ1-42 oligomers) with respect to resting cells or cells exposed to Aβ1-42 oligomers (*p* < 0.05; Figure 7B). When the incubation time was prolonged to 24 h, expression levels of *PTGS2* in cells treated with carnosine alone were significantly decreased compared to resting cells (*p* < 0.05).

The mRNA levels of the antioxidant enzymes glutathione reductase (GSR) and superoxide dismutase (SOD) 1 and 2 were less affected in our experimental conditions (Figure 7C). One-way ANOVA failed to reveal a main effect for *GSR* and *SOD2* at both time points. Exposure for 6 h to Aβ1-42 oligomers caused a small but significant increase in *SOD1* mRNA (by about 10%) compared to resting cells or cells exposed to Aβ1-42 oligomers and carnosine (*p* < 0.05).

### 3.7. Carnosine Enhanced the Phagocytic Activity of Macrophages

**Figure 8:**
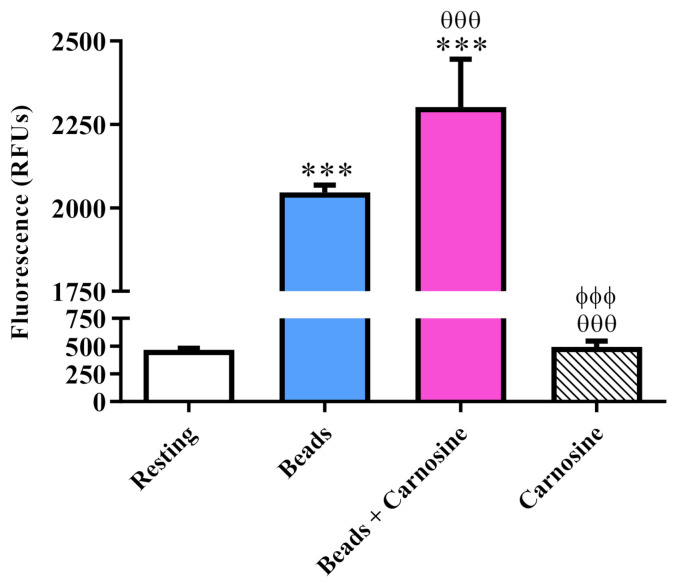
Measurement of macrophage phagocytic activity in resting RAW 264.7 cells and in RAW 264.7 cells stimulated with antibody-bound tentagel beads in the absence or presence of carnosine (20 mM; 1 h pre-treatment). Data are the mean of 3 to 8 independent experiments and are expressed as relative fluorescence units (RFUs). Standard deviations are represented by vertical bars. *** significantly different from resting cells, *p* < 0.001; ^ѲѲѲ^ significantly different from bead-treated cells, *p* < 0.001; ^ϕϕϕ^ significantly different from beads + carnosine-treated cells, *p* < 0.001.

Given that carnosine has been shown to increase the phagocytic activity of macrophages in vivo [35,63], we wondered whether the well-known antioxidant activity of carnosine was also paralleled by the ability of this dipeptide to increase the phagocytic activity of RAW 264.7 cells. In order to validate this hypothesis, we used a recently developed protocol that allowed us to measure phagocytosis in RAW 264.7 cells by using antibody-bound tentagel beads and PS3 [52,53]. As clearly depicted in Figure 8, the addition of antibody-opsonized beads to RAW 264.7 macrophages resulted in a significant increase of cellular fluorescence compared to resting cells (*p* < 0.001).

The presence of carnosine during the addition of the functionalized beads further increased the fluorescence signal (*p* < 0.001 compared to resting cells or beads-treated cells), suggesting an enhanced ability of RAW 264.7 macrophages to phagocytize the beads. In order to strengthen this hypothesis, we also treated the cells with carnosine only, allowing us to prove that the increase in fluorescence signal in the beads + carnosine sample was not due to carnosine per se; in fact, as shown in Figure 8, no significant differences were observed between resting and carnosine-treated RAW 264.7 cells.

## 4. Discussion

Carnosine is a naturally occurring endogenous dipeptide. It is characterized by well-known direct and indirect antioxidant activities that include the clearance of ROS and RNS [64], along with anti-aggregation [65,66] and anti-inflammatory [67] effects. These effects suggest potential therapeutic applications of this molecule for the treatment of neurodegenerative disorders characterized by oxidative stress, abnormal protein aggregation, and neuroinflammation such as AD [33].

It is now well-known that the oligomeric forms of Aβ1-42 peptide represent the most toxic species of Aβ. These oligomers lead to synaptic loss and neuronal death in the brain of AD subjects [68]. Aβ can induce neuronal death by acting directly on neurons or by stimulating the production of inflammatory and toxic factors from microglia or from infiltrating mononuclear cells [69]. A pivotal role mediating the toxicity of Aβ oligomers is played by oxidative stress; in fact, on one hand, Aβ oligomers have been shown to impair synaptic plasticity and promote neurodegeneration and neuroinflammation via oxidative stress [70,71], whereas on the other hand, oxidative stress promotes the oligomerization of Aβ peptide [72].

Both microglia and neuronal cultures treated with Aβ1-42 oligomers provide a widely accepted model of inflammation and neurodegeneration occurring in AD pathology [73,74,75]. Recently, the use of macrophages, in particular RAW 264.7 cells, to study the toxic effects of the aggregated form of Aβ as well as the therapeutic potential of antioxidants has been considered [47,76]. The interest in this type of study is also driven by the fact that, as previously mentioned, infiltrating macrophages have been shown to protect from Aβ toxicity by clearing this peptide from the brain through its uptake and subsequent degradation [20,21]. The key role of peripheral monocytes/macrophages in the pathophysiology of AD is also reinforced by a report that a defective ability of these cells to engulf Aβ is observed in AD patients [22]. Along this line, the ability of carnosine to modulate the activity of immune cells such as macrophages and microglia [36,49,75,76], including the enhancement of their antioxidant machinery [49] as well as the increased production of anti-inflammatory molecules such as TGF-β1 [36], could be highly relevant for drug development in AD.

According to this scenario, in the present study, we first explored the toxicity induced by Aβ1-42 oligomers on RAW 264.7 macrophage cells. When monitoring cellular toxicity under our experimental conditions, we observed that the treatment with Aβ1-42 oligomers significantly increased the number of dead macrophages (Figure 1). Figure 1 also depicts the ability of carnosine to significantly decrease the toxicity induced by Aβ. We hypothesized that these protective effects could be related to the ability of carnosine to counteract oxidative stress in macrophage cells [49,50]. The protective effects of carnosine were corroborated by the results presented in Figure 2, showing that the presence of carnosine during treatment with toxic and pro-apoptotic Aβ1-42 oligomers decreased the percentage of cell population undergoing apoptosis.

We then examined the correlation between the toxicity induced by Aβ oligomers and the production of NO (a component of RNS) and ROS, two well-known “contributors” to neurodegenerative phenomena observed in AD [77,78]. Both NO and RNS, increased as a consequence of treatment with Aβ1-42 oligomers and were significantly diminished in the presence of carnosine (Figure 3 and Figure 4). This is consistent with the well-known antioxidant power of this dipeptide associated with (1) its ability to directly interact with these species [79], and (2) the presence of the imidazole ring of histidine [37]. With regard to the observed decrease in NO intracellular levels, it is not necessarily evidence of decreased NO production in macrophages; in fact, as shown in two previous studies employing RAW 264.7 cells pre-treated with carnosine (1 h) and then stimulated with LPS, carnosine did not change the production of NO, but instead increased the degradation rate of this molecule into the non-toxic end product nitrite, therefore decreasing NO availability [37,49]. The results showing the ability of carnosine to reduce species related to oxidative stress phenomena are in accordance with other studies in which carnosine protected neuronal cells against oxidative stress through the modulation of MAPK pathway [80] or exerted neuroprotection in primary culture of rat cerebellar cells challenged with 2,2′-azobis(2-amidinopropane) dihydrochloride, or rotenone, treatments that generate free radicals [81]. Of note, the results presented in this study are also in line with a study carried out by Corona et al., showing that carnosine supplementation in 3xTg-AD mice, a transgenic model of AD, led to a strong reduction in the hippocampal intraneuronal accumulation of Aβ and completely rescued AD and age-related mitochondrial dysfunction [82].

The observed decrease in toxicity and apoptosis as well as of NO and ROS levels could be the consequence of an increased loading of carnosine by RAW 264.7 cells activated under stress conditions [51], of the ability of carnosine to increase the rate of conversion of NO into its non-toxic end-product nitrite [37], and/or of the ability of this endogenous dipeptide to preserve the monomeric form of Aβ peptide or to disassemble the Aβ aggregates already formed [83,84].

With the present study, we were also able to demonstrate that levels of peroxynitrite, a more dangerous species compared to NO or ROS [85], are significantly induced in macrophages following stimulation with Aβ1-42 oligomers, whereas are reduced upon pre-treatment with carnosine (Figure 5). The increase in peroxynitrite levels in the presence of Aβ could be associated with the engulfment of Aβ peptide [20,21], since an increase in production of endogenous peroxynitrite has been demonstrated during phagocytosis by macrophages [52].

Given that Aβ toxicity appears to be highly interconnected with the inflammatory response [86], we then investigated whether the protective effects of carnosine may be mediated through modulation of the expression of markers of inflammation, such as cytokines. Consistent with report by other groups [37,87], an inflammatory stimulus such as LPS, one of the main components of the outer membrane of Gram-negative bacteria, induced a strong and persistent upregulation of the expression levels of *TNF-α*, *IL-6*, *IL-1β*, *NOS2*, and *PTGS2* in RAW 264.7 cells. However, in cells exposed to Aβ1-42 oligomers (6 or 24 h) the expression levels of *TNF-α*, *IL-1β*, and *IL-6*, as well as of *IL-10*, *TGF-β1*, and *CXCL2* were not affected with the same relevant extent observed in LPS-stimulated cells (Figure 6; Figure S1 and Supplementary Results 1). Gene expression analysis revealed that *IL-6*, *IL-1β*, *TGF-β1*, and *IL-10* were not changed in our experimental conditions, whereas *TNF-α* and *CXCL2* were increased by a short-term exposure to Aβ1-42 oligomers. The transcriptional effects of Aβ1-42 oligomers on *TNF-α* and *CXCL2* were dampened when macrophages were pre-treated with carnosine, which when administered alone increased the expression of these targets following a 6-h exposure. CXCL2 is among the most effective neutrophil chemoattractants and its expression is induced by several stimuli including LPS or Aβ peptides both in vitro and in vivo [88,89]. Future studies will be needed to better elucidate the impact of carnosine on Aβ-induced effects on these complex systems. Cytokine signaling, in fact, is extremely redundant [90] and tightly regulated. What is observed at the transcriptional level may not be mirrored by protein levels or cytokine release. In fact, it has been recently demonstrated in BV2 cells that co-incubation with carnosine counteracted the effects of Aβ1-42 oligomers on the release of specific cytokines, without affecting their expression levels [36].

One of the most surprising findings was that a short exposure to carnosine increased the expression levels of *NOS2* in RAW 264.7 cells. This effect lasted at least up to 24 h in cells treated with Aβ1-42 oligomers in presence of carnosine (Figure 7). Again, the observed induction under our experimental conditions was significantly less pronounced than that observed following exposure to LPS (3-fold in response to Aβ + carnosine or carnosine alone, 500-fold in response to LPS). In previous experiments, it was demonstrated that carnosine dose-dependently increased the concentration of intracellular nitrite in macrophages stimulated with LPS; however, this increase did not affect total NO production induced by endotoxin stimulation [37]. NO is a crucial mediator in pathology and physiology [91]; given that RAW 264.7 cells did not express the constitutive isoform of NO synthase (data not shown), a transient upregulation of *NOS2* induced by carnosine alone may represent a step to activate a NO-mediated signaling cascade. In endothelial cells, it was demonstrated that carnosine at concentrations higher than 5 mM facilitated NO formation by increasing intracellular calcium mobilization and activation of endothelial NOS [92]. In immune cells, NO may as well serve to limit the extent of potentially destructive immune responses, and increased NO production has been correlated to initial increases in microglial phagocytosis [93,94,95]. Given that carnosine increases the phagocytic activity of macrophages in vivo [63], in addition to its antioxidant and free-radical scavenging roles, this possible link was also investigated. By applying a recently developed protocol that uses a fluorescent sensor targeted to membranes of the endoplasmic reticulum and allowing for the assessment of phagocytosis by measuring the related peroxynitrite formation, we were able to show that carnosine enhances the phagocytic activity of RAW 264.7 macrophages (Figure 8). Our results are in accordance with two previous studies carried out by Li et al. [96] and Rios et al. [97], demonstrating an enhanced peroxynitrite production during the process of phagocytosis. The above-mentioned studies along with the one published by Alvarez et al. [98], showing the intraphagosomal formation of peroxynitrite as a macrophage-derived cytotoxin against internalized *Trypanosoma cruzi*, could also explain the transient activation of pro-oxidant enzymes such as NOS2 observed in the presence of carnosine. Despite the protective effect of carnosine on Aβ-induced increase in the production of NO and ROS levels, the expression levels of *GSR*, *SOD1*, and *SOD2* antioxidant enzymes were not significantly modulated by the treatments with Aβ, Aβ + carnosine, or carnosine alone (with the sole exception of a small transient induction of SOD1 at 6 h). Interestingly, carnosine rescued the downregulation of the expression of the fractalkine receptor *CX3CR1* mediated by Aβ1-42 oligomers after 24 h (Figure 6). CX3CR1, the exclusive receptor for the chemokine fractalkine (CX3CL1), is expressed by several cell types, including monocytes in the circulation and by microglia in the central nervous system [99]. As expected, RAW 264.7 cells did not express *CX3CL1*, whereas *CX3CR1* mRNA was abundantly expressed in resting cells. Here, we observed that, despite a different timing, downregulation of *CX3CR1* was a common outcome of exposure to both LPS (6 h) or Aβ1-42 oligomers (24 h) (Figure 6; Figure S1 and Supplementary Results 1). Our data suggest that carnosine may affect the functionality of the CX3CL1/CX3CR1 axis, a key regulator of neuron–microglia interaction in AD pathology. Disruption of the fractalkine signaling by deletion of the CX3CR1 receptor may cause microglia to proliferate faster and cluster around fibrillar amyloid plaques [100,101]. Data from AD mouse models suggest its involvement in the modulation of neuronal survival, plaque load, and cognition [102,103,104]. The rescue of this receptor by carnosine could be of great relevance for future drug discovery processes in AD since it has been shown that the absence of CX3CR1 impairs the internalization of toxic tau by brain macrophages, thus favoring an accumulation of extracellular tau, a key event in the progression of AD [105]. Evidence suggests that this system, however, may have complex interactions with the hallmark pathologies of AD and may exert neuroprotective or neurotoxic effects depending on the state of disease progression [106]. Rescue of CX3CR1 by carnosine might therefore represent a novel mechanism that can contribute to the overall neuroprotective activity of this peptide against Aβ1-42 oligomer toxicity, but further studies are needed in experimental models of AD.

## 5. Conclusions

In the present study, we were able to show that carnosine suppresses cell death and apoptosis induced by Aβ1-42 oligomers in RAW 264.7 macrophages by decreasing oxidative stress. In particular, this dipeptide decreased intracellular NO and ROS levels and counteracted the production of peroxynitrite. This protective activity of carnosine was not mediated by the modulation of the canonical inflammatory pathway but is proposed to involve the well-known free-radical scavenging activity of this dipeptide, enhanced phagocytic activity, and the rescue of fractalkine receptor CX3CR1.

Inhibition of oxidative stress mediated by Aβ1-42 oligomers and the rescue of the fractalkine receptor CX3CR1 have been recently considered as novel therapeutic strategies to prevent neuronal loss and cognitive decline in AD pathology. Our results indicate that the multimodal dipeptide carnosine has a therapeutic potential as a new pharmacological tool for modulating these targets in the context of AD pathology.

## Data Availability

The data presented in this study are available on request from the corresponding author.

## Supplementary Materials

The following are available online at https://www.mdpi.com/article/10.3390/biomedicines9050477/s1, Supplementary Results 1 and Figure S1: Effects of exposure of RAW 264.7 cells to LPS for 6 and 24 h on expression levels of immune-related targets; Table S1: Nucleotide sequence of the forward and reverse primers used for qRT-PCR.
