# Supplementary material for: Carnosine Protects Macrophages against the Toxicity of Aβ1-42 Oligomers by Decreasing Oxidative Stress
Source: Biomedicines. 2021 Apr 26;9(5):477. doi: 10.3390/biomedicines9050477 (PMC8146816; doi:10.3390/biomedicines9050477)
Supplement: Supplementary file 1 [file biomedicines-09-00477-s001.zip › biomedicines-1188201-supplementary.pdf]

biomedicines

Supplementary Material

Supplementary results

Effects of exposure of RAW 264.7 cells to LPS for 6 and 24 h on expression levels of immune-related targets. These experiments were used to confirm that the expression of markers of immune activation in RAW 264.7 was strongly responsive to an immune challenge such as LPS. Cells were exposed to LPS (100 ng) and harvested after 6 or 24 h.

As expected, *IL-1β*, *IL-6*, and *TNF-α* were significantly up-regulated in RAW 264.7 cells exposed to the endotoxin for 6 h: the increase for *IL-6* and *IL-1β* mRNA was more than 45000-fold, whereas *TNF-α* was increased by LPS by ~20 fold as compared to resting cells (*IL-1β*: $p < 0.01$; *IL-6*: $p < 0.001$; *TNF-α*: $p < 0.01$). The chemokine *CXCL2* was significantly increased as well in cells exposed to LPS for 6 h: the mRNA coding for one of the murine functional homologs of human CXCL8 was increased more than 1700-fold compared to resting cells harvested at the same time ($p < 0.001$). *TGF-β1* mRNA levels were increased by almost 3-fold in LPS exposed cells compared with resting cells under the same experimental conditions ($p < 0.001$).

After a 24-h exposure to the endotoxin, the expression of *IL-1β*, *IL-6*, *TNF-α*, *CXCL2*, and *TGF-β1* was still significantly higher compared to resting cells. The magnitude of the increase, however, was lower than that observed after 6 h: *IL-6* and *IL-1β* were increased by about 10000-fold (*IL-1β*: $p < 0.01$; *IL-6*: $p < 0.001$) *TNF-α* by about 2-fold ($p < 0.001$), *CXCL2* by about 190-fold ($p < 0.001$), *TGF-β1* by 0.2-fold ($p < 0.01$) compared to resting RAW 264.7 cells. IFN-γ mRNA levels remained undetected in RAW 264.7 cells even in presence of immune stimulation.

The mRNA levels of *CX3CR1* were instead significantly decreased following a 6-h exposure to LPS compared to resting cells ($p < 0.001$). This effect was no longer present after 24 h, where the expression levels of the fractalkine receptor were not statistically different between stimulated and resting cells.

An up-regulating effect of LPS exposure in RAW 264.7 cells was also observed for transcription of *NOS2* and *PTGS2*. After a 6 h exposure, LPS induced *NOS2* mRNA by about 500-fold, and *PTGS2* mRNA by about 1500-fold compared to resting cells ($p < 0.001$). At the 24-h time point, the expression levels of *NOS2* in stimulated cells remained of the same order of magnitude observed at the earlier time point, whereas *PTGS2* mRNA levels continued to increase by over 2000-fold compared to resting cells harvested at the same time (*NOS2*: $p < 0.001$; *PTGS2*: $p < 0.001$).

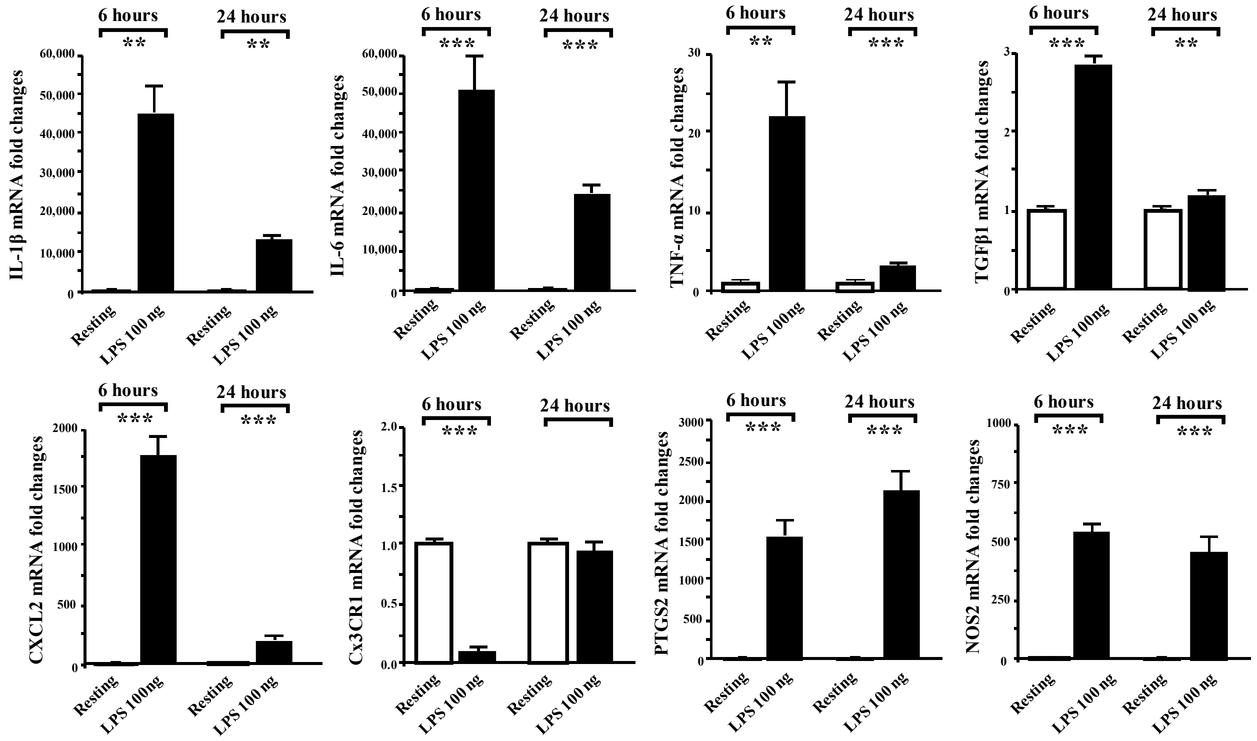

Figure S1. Effects of exposure of RAW 264.7 cells to LPS for 6 and 24 h on expression levels of immune-related targets in RAW 264.7 cells. Cell were left untreated (control) or treated with LPS (100 ng/mL) for 6 and 24 h after which mRNA expression of Interleukin (IL)-1β, IL-6, Tumor Necrosis Factor (TNF)-α, Transforming-Growth-Factor (TGF)-β1, Macrophage Inflammatory Protein-2 (CXCL2), CX3CR1, inducible nitric oxide synthase (NOS2), and Prostaglandin-Endoperoxide Synthase 2 (PTGS2), with GAPDH/CypA as endogenous control, were measured by qRT-PCR. Data are represented as means ($n = 4$) ± standard error of the mean (S.E.M.). **significantly different from resting cells, $p < 0.01$, ***significantly different from resting cells, $p < 0.001$.

Table S1. Nucleotide sequence of the forward and reverse primers used for qRT-PCR.

Target Name	Primer Sequence (5′-3′)	Gene Bank Number	Amplicon Length	Mean Cq in Resting Cells
IL-6	Fw: CTTCACAAGTCGGAGGCTTA<br>Rv: CAAGTGCATCATCGTTGTTC	NM_031168.2	107<br>(nt 221–328)	*38.3*
IFN-γ	Fw: TCTTCAGCAACAGCAAGGCGAA<br>Rv: ACAGCTGGTGGACCACTCGGA	NM_008337.4	119<br>(nt 414–533)	*Undetectable*
IL-1β	Fw: TGAAAGCTCTCCACCTCAATG<br>Rv: CCAAGGCCACAGGTATTTTG	NM_008361.4	104<br>(nt 515–619)	30.8
TNF-α	Fw: GGCCTCCCTCTCATCAGTTC<br>Rv: CACTTGGTGGTTTGCTACGA	NM_013693.3	104<br>(nt 366–470)	**21.1**
IL-10	Fw: GAAGCATGGCCCAGAAATCAAG<br>Rv: AAATCACTCTTCACCTGCTCCAC	NM_010548.2	140<br>(nt 364–504)	30.6
CXCL2	Fw: TCAATGCCTGAAGACCCTGC<br>Rv: TTTGACCGCCCTTGAGAGTG	NM_009140.2	118<br>(nt 154–272)	**23.4**
CXCL1	Fw: TGGCTGGGATTCACCTCAAG<br>Rv: CAAGCCTCGCGACCATTCTT	NM_008176.3	108<br>(nt 182–290)	34.8
TGF-β1	Fw: CAAGGGCTACCATGCCAACTT<br>Rv: GTTGTGTTGGTTGTAGAGGGC	NM_011577.2	100<br>(nt 1809–1909)	**20.4**
CX3CL1	Fw: CTTCCATTTGTGTACTCTGCTGC<br>Rv: GACTCCTGGTTTAGCTGATAGCG	NM_009142.3	125<br>(nt 130–255)	*36.2*
CX3CR1	Fw: CGTGAGACTGGGTGAGTGACT	NM_009987.4	125	**20.2**

	Rv: TCAGCAGAATCGTCATACTCAAA		[nt 35–160]	
NOS2	Fw: ACGAGACGGATAGGCAGAGA<br>Rv: GAGTAGTAGCGGGGCTTCAA	NM_010927.4	183<br>(nt 3201–3384)	**28.7**
PTGS2	Fw: TTCTACGGAGAGAGTTCATC<br>Rv: CAGTTTATGTTGTCTGTCCA	NM_011198.4	187<br>(nt 696–883)	**25.5**
Gsr	Fw: CTATGACAACATCCCTACTG<br>Rv: GTAAAGGCAGTCGAGTAGAT	NM_010344.4	122<br>(nt 1457–1579)	**21.7**
Sod1	Fw: GGGAAGCATGGCGATGAAAG<br>Rv: CCCCATACTGATGGACGTGG	NM_011434.2	163<br>(nt 90–253)	**19.7**
Sod2	Fw: CTTTTGAGAAGTTTAAGGAGA<br>Rv: TGACGTTTTTATACTGAAGG	NM_013671.3	209<br>(nt 587–796)	**21.4**
CypA	Fw: AGCATACAGGTCCTGGCATC<br>Rv: TTCACCTTCCCAAAGACCAC	NM_008907.2	122<br>(nt 316–442)	**17.27**
GAPDH	Fw: CAAGGTCATCCATGACAACTTTG<br>Rv: GGGCCATCCACAGTCTTCTG	NM_008084.3	89<br>(nt 717–806)	**16.83**

The accession number and the size (bp) of the PCR product obtained by amplification of the cDNA (mRNA) are given for each target. As an indication of the relative abundances of each target, average Cq values for resting cells are provided (20 ng; n = 18–26). Transcripts with an average Cq value of ≥35 were considered as low expression genes (Italic), genes with Cq values between 30 and 34 were of medium expression, and those with Cq values of ≤29 were highly expressed (Bold). IL: interleukin; TNF: tumor necrosis factor; IFN: interferon; TGF: transforming growth factor; NOS: NO synthase; PTGS: prostaglandin-endoperoxide synthase; GSR: glutathione reductase; SOD: superoxide dismutase; CypA: peptidylprolyl isomerase A; GAPDH: glyceraldehydes-3-phosphate dehydrogenase.
